# Longitudinal Associations of Stroke With Cognitive Impairment Among Older Adults in the United States: A Population-Based Study

**DOI:** 10.3389/fpubh.2021.637042

**Published:** 2021-05-19

**Authors:** Xia Wu, Li Fan, Songqing Ke, Yangting He, Ke Zhang, Shijun Yang

**Affiliations:** ^1^Department of Otorhinolaryngology, Union Hospital, Tongji Medical College, Huazhong University of Science and Technology, Wuhan, China; ^2^Department of Orthopaedics, Union Hospital, Tongji Medical College, Huazhong University of Science and Technology, Wuhan, China; ^3^Ministry of Education Key Laboratory of Environment and Health, Department of Epidemiology and Biostatistics, School of Public Health, Tongji Medical College, Huazhong University of Science and Technology, Wuhan, China; ^4^Biostatistician at Causality Clinical Data Technology Co., Ltd, Wuhan, China; ^5^Department of Cardiology, Union Hospital, Tongji Medical College, Huazhong University of Science and Technology, Wuhan, China

**Keywords:** stroke, cognitive decline, older, longitudinal analysis, United States

## Abstract

**Objective:** The aim of this study was to explore the longitudinal associations of stroke with cognitive impairment in older US adults.

**Method:** The data used in this longitudinal analysis were extracted from the National Health and Aging Trends Study (NHATS) from 2011 to 2019. Univariate and multivariable Cox proportional hazards regression models were used to estimate the longitudinal association of stroke with cognitive impairment. The multivariable model was adjusted by demographic, physical, and mental characteristics, and the complex survey design of NHATS was taken into consideration.

**Results:** A total of 7,052 participants with complete data were included. At the baseline, the weighted proportion of cognitive impairment was 19.37% (95% CI, 17.92–20.81%), and the weighted proportion of the history of stroke was 9.81% (95% CI, 8.90–10.72%). In univariate analysis, baseline stroke history was significantly associated with cognitive impairment in the future (hazard ratio, 1.746; 95% CI, 1.461–2.088), and the baseline cognitive impairment was significantly associated with future report of stroke (hazard ratio, 1.436; 95% CI, 1.088–1.896). In multivariable model, stroke was also significantly associated with cognitive impairment (hazard ratio, 1.241; 95% CI, 1.011–1.522); however, the reverse association was not significant (hazard ratio, 1.068; 95% CI, 0.788–1.447). After the data from proxy respondents were excluded, in the sensitive analyses, the results remained unchanged.

**Conclusion:** Older adults in the United States who suffered strokes are more likely to develop cognitive impairment as a result in the future than those who have not had strokes. However, the reverse association did not hold. Furthermore, the study suggests that it is necessary to screen and take early intervention for cognitive impairment in stroke survivors and prevent the incidence of stroke by modifying risk factors in the general population with rapidly growing older US adults.

## Introduction

Stroke, described as an interruption to the supply of blood to the brain by WHO (1), is one of the leading causes of death and disability in the United States (2). An estimated 7.2 million Americans self-reported having strokes until 2014 based on data from the National Health and Nutrition Examination Survey (NHANES) 2011–2014. Despite the decreasing trend for the incidence of stroke in the United States, the aging population leads to a higher risk of stroke through their lifetime. Dementia is a clinical disorder caused by neurodegeneration, the main manifestation of which is progressive multiple cognitive impairment (3). According to the results of 2015 Alzheimer's Disease Facts and Figures, there is a dramatic growing trend of dementia, and the prevalence is estimated to be growing to nearly 15 million by 2050 in the United States (4). Both stroke and dementia have posed a heavy burden on the health system and serious influence on economies in the United States (5).

Stroke and dementia often co-occur (6), and most cases occur in people aged 65+ years (7, 8). A large number of studies have revealed that cognitive impairment was more likely to occur in stroke survivors than non-stroke individuals either in or outside the United States (9–18). However, the studies were often limited in small sample size or old data. On the other hand, the reverse association (the risk of stroke development after cognitive impairment prognosis) was less investigated, and the results were controversial (19–23). In addition, only one study conducted in Canada has examined the bidirectional relationship of stroke development and cognitive impairment at the same time (22).

In this study, we collected data from a national representation of the older adult cohort used as the study sample in the United States during 2011–2019. And we investigated the longitudinal bidirectional association between self-reported stroke and cognitive impairment in a relatively long-term period to provide the latest evidence for the design of target disease prevention and health interventions in older US adults.

## Materials and Methods

### Study Sample

We used data from round 1 (2011) to round 9 (2019) of the National Health and Aging Trends Study (NHATS) data sets, a cohort of US sample adults aging 65 years and older who received Medicare (24). The NHATS has collected the information about their mental and physical function annually by in-person interviews to investigate the trends in late life since 2011. The up-to-date data were collected in 2019. Our study included the follow-up data over 8 years. The baseline in our study was defined as the status in round 1. A total of 8,245 participants were included in round 1. The NHATS survey protocol was approved by the Johns Hopkins University Institutional Review Board, and all participants provided informed consent.

### Measures

#### Stroke

The status of stroke in baseline was determined by the answers of participants who were asked if they were diagnosed as having a stroke by a physician or not.

#### Cognitive Impairment

In NHATS, the cognition of participants was measured in three domains that measured memory, orientation, and executive functioning (25). The cognitive impairment classification was divided into three levels, including probable cognitive impairment, possible cognitive impairment, and no cognitive impairment, which were based on the criteria of the NHATS protocol. We defined the probable cognitive impairment and possible cognitive impairment as cognitive impairment. The probable cognitive impairment was determined by three ways: the report diagnosed as dementia or Alzheimer's disease by clinical physician; the AD8 score at or higher than 2 if proxy respondents did not report the diagnosis of cognition; or scores at or below 1.5 standard deviations (SDs) less than the mean value of at least two domains of cognitions. The score at or below 1.5 SD less than the mean in one cognitive domain indicated possible cognitive impairment. The other participants who were not assessed to have probable or possible cognitive impairment were defined as no cognitive impairment.

### Covariates

In this study, the covariates we included were age, gender, race/ethnicity, educational level, Medicaid eligibility, body mass index (BMI), smoking status (never, former, and current), the number of comorbidities (heart attack, hypertension, heart disease, arthritis, osteoporosis, diabetes, lung disease, and cancer), and the need for a proxy respondent. The anxious and depressive symptoms were determined by the scale of the Patient Health Questionnaire for Depression and Anxiety (PHQ-4) (26). The PHQ-4 included two subscales: the PHQ-2 measuring depression (27) and the Generalized Anxiety Disorder-2 (GAD-2) measuring anxiety (28). In PHQ-2, participants who scored 4–6 were defined as having a depressive symptom. In GAD-2, participants who scored 4–6 were defined as having an anxious symptom.

### Statistical Analysis

The weighted proportions of the baseline characteristics for participants were calculated, and the participants were stratified by their cognitive and stroke status in the 8 years' follow-up. The descriptive comparisons were performed using the Rao–Scott chi-square test for categorical variables and the unpaired *t*-tests for continuous variables. The longitudinal association of stroke with cognitive impairment was estimated by univariate and multivariable Cox proportional hazards regression model. In the first set of models, we used the stroke status at the baseline to predict cognitive impairment in the future. And in the second set of models, the cognitive status at the baseline was to estimate its impact on the stroke status in the future. Participants with the outcome of interest at the baseline were excluded in the corresponding analysis. In the first set of models, we excluded the participants who had been having a stroke at the baseline. In the second set of models, we excluded the participants who had been having cognitive impairment at the baseline. The censor was identified as the participants who were lost to follow-up, had never reported an event, or had died. Besides, the Kaplan–Meier method was used to estimate the unadjusted association of stroke with cognitive impairment. Furthermore, we excluded the data from proxy respondents to perform a sensitivity analysis. The complex sampling design of NHATS was taken into account in our analyses. All statistical analyses were performed using SAS 9.4 (SAS Institute Inc., Cary, North Carolina, USA) and R 3.6.1 (R Foundation for Statistical Computing, Vienna, Austria). Two-sided *P* < 0.05 was statistically significant.

## Results

At the baseline, after the participants with missing value of one or more variables were excluded, 7,052 participants were included in our study. Of the 7,052 participants, 1,762 had cognitive impairment at the baseline and accounted for 19.37% (95% CI, 17.92–20.81%), and 808 had a history of stroke at the baseline (9.81%; 95% CI, 8.90–10.72%). The survey-weighted proportion of participants was 56.09% for females (95% CI, 54.68–57.50%) and 53.45% for participants aged 65–74 (95% CI, 51.39–55.52%).

After 1,762 participants with cognitive impairment at the baseline were excluded, as Table 1 shows, compared with those with no cognitive impairment in the 8 years of follow-up, the participants with cognitive impairment were more likely to be older, to be non-white, have lower educational level, have dual Medicare–Medicaid eligibility, need a proxy respondent, to be more depressive, to be more anxious, have lower BMI, have more comorbidities, and smoke less. Importantly, the weighted proportion of the history of stroke was higher in respondents with cognitive impairment (11.39%; 95% CI, 9.33–13.46%) than those with no cognitive impairment in the follow-up (7.00%; 95% CI, 6.17–7.83%).

**Table 1:** Weighted sample characteristics and comparisons stratified by cognitive impairment during the 8 years of follow-up^a^.

**Characteristic**	**Cognitive impairment**		***P^c^***
	**Yes^b^ (*N* = 1,458) (95% CI)**	**No (*N* = 3,832) (95% CI)**	
**Age groups, %, year**			<0.0001
65–69	17.00 (14.40, 19.60)	36.73 (34.94, 38.51)	
70–74	24.29 (21.48, 27.11)	27.68 (26.29, 29.07)	
75–79	22.32 (19.74, 24.90)	18.14 (17.02, 19.25)	
80–84	19.46 (16.89, 22.03)	11.19 (10.28, 12.10)	
85–89	12.39 (10.63, 14.14)	4.68 (4.04, 5.32)	
≥90	4.53 (3.58, 5.49)	1.59 (1.27, 1.90)	
**Sex, %**			0.3352
Male	42.23 (39.42, 45.03)	43.84 (42.00, 45.68)	
Female	57.77 (54.97, 60.58)	56.16 (54.32, 58.00)	
**Race/ethnicity, %**			<0.0001
White, non-hispanic	79.09 (74.71, 83.47)	86.18 (83.76, 88.59)	
Black, non-hispanic	9.82 (7.32, 12.33)	6.57 (5.07, 8.07)	
Hispanic	8.21 (5.02, 11.40)	4.49 (3.06, 5.93)	
Other	2.87 (1.59, 4.15)	2.76 (1.97, 3.56)	
**Educational level, %**			<0.0001
No degree	25.55 (22.25, 28.85)	13.37 (11.75, 15.00)	
High school	36.50 (33.50, 39.49)	35.58 (32.84, 38.32)	
Some college	12.61 (10.46, 14.76)	16.07 (14.57, 17.56)	
College degree and more	25.35 (21.81, 28.89)	34.98 (31.88, 38.07)	
**Medicare-Medicaid enrollees, %**			<0.0001
Yes	13.57 (10.89, 16.26)	7.47 (6.41, 8.53)	
No	86.43 (83.74, 89.11)	92.53 (91.47, 93.59)	
**Proxy respondent, %**			0.0023
Yes	2.29 (1.45, 3.13)	1.09 (0.71, 1.46)	
No	97.71 (96.87, 98.55)	98.91 (98.54, 99.29)	
**Depression, %**			<0.0001
Yes	7.17 (5.41, 8.93)	3.75 (3.15, 4.36)	
No	92.83 (91.07, 94.59)	96.25 (95.64, 96.85)	
**Anxiety, %**			<0.0001
Yes	10.01 (8.12, 11.90)	4.78 (4.02, 5.54)	
No	89.99 (88.10, 91.88)	95.22 (94.46, 95.98)	
**Smoking status, %**			0.0022
Never	49.37 (46.55, 52.18)	45.18 (43.02, 47.34)	
Former	42.57 (39.74, 45.40)	45.64 (43.56, 47.73)	
Current	8.06 (6.13, 9.99)	9.17 (8.01, 10.34)	
Stroke, %	11.39 (9.33, 13.46)	7.00 (6.17, 7.83)	<0.0001
Comorbidities, mean	3.96 (3.87, 4.05)	3.72 (3.67, 3.77)	<0.0001
Body mass index, mean	32.61 (32.18, 33.05)	33.24 (32.96, 33.53)	0.0096

a *Excluding participants with cognitive impairment at the baseline (N = 1,762)*.

b *Includes participants classified as probable and possible cognitive impairment*.

c *P-values are calculated by the Rao-Scott Chis-square test for categorical variables and the unpaired t-tests for continuous variables*.

**Figure 1:**
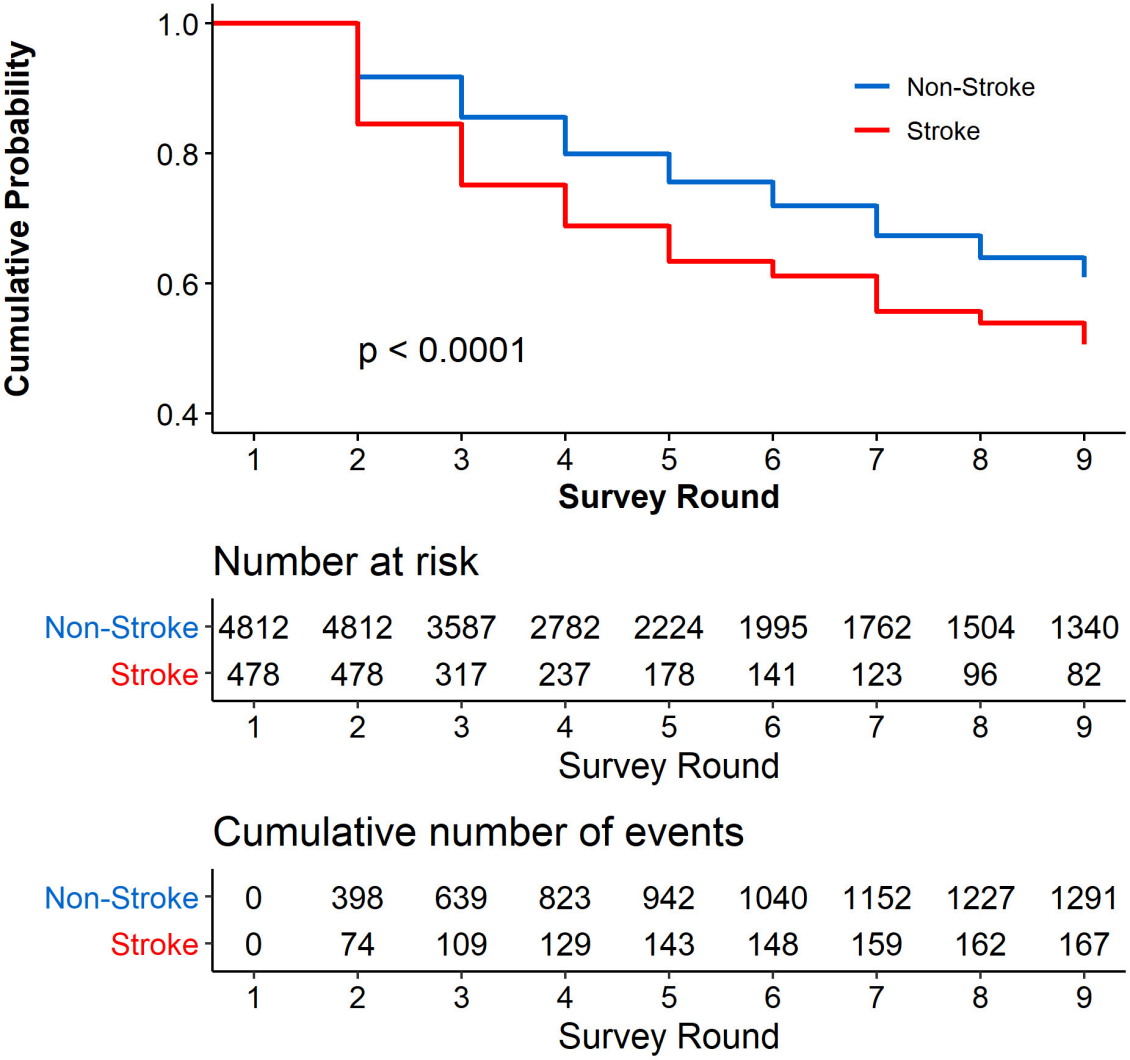
Cumulative Probability of not reporting cognitive impairment during the 8 years of follow-up. Respondents with cognitive impairment at the baseline were excluded (*N* = 1,762).

Figure 1 shows the cumulative probability of not reporting cognitive impairment during the 9 years of follow-up among the participants stratified by stroke and non-stroke at the baseline. The cumulative proportion for participants with stroke who did not report cognitive impairment was 50.68% (95% CI, 44.53–56.83%), and for those with non-stroke who did not report cognitive impairment, the cumulative proportion was 60.93% (95% CI, 59.13–62.73%). We found that the cumulative proportion of not reporting cognitive impairment was significantly higher in respondents with non-stroke than those with stroke at the baseline (*P* < 0.0001). Supplementary Table 1 shows the results of univariate Cox proportional hazards regression; stroke at the baseline was significantly associated with cognitive impairment [hazard ratio (HR), 1.746; 95% CI, 1.461, 2.088; *P* < 0.0001].

**Figure 2:**
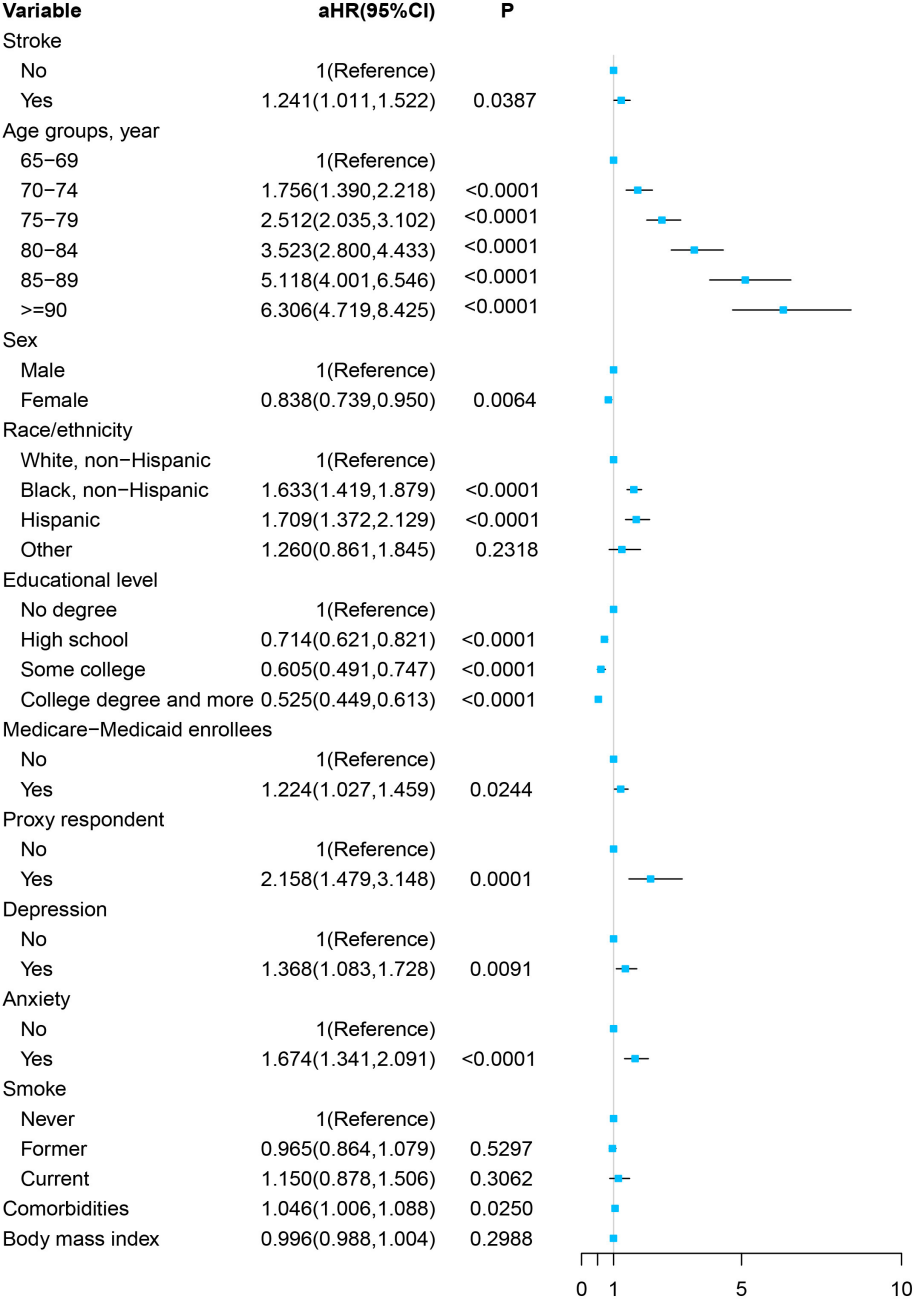
Forest plot of multivariable proportional hazards regression modeling the development of cognitive impairment over 8 years of follow-up.

As Figure 2 and Supplementary Table 2 show, in the multivariable Cox proportional hazards regression model, adjusted by the age, sex, race/ethnicity, educational level, Medicare–Medicaid eligibility, proxy respondent, depression, anxiety, smoking status, comorbidities, and BMI, the participants with stroke at baseline were more likely to develop cognitive impairment in the future significantly [adjusted HR (aHR), 1.241; 95% CI, 1.011–1.522, *P* = 0.0387].

After 808 participants with the history of stroke at the baseline were excluded, as Table 2 shows, compared with those with no stroke symptom in the 8 years of follow-up, the participants with stroke were more likely to be older, have dual Medicare–Medicaid eligibility, and have more comorbidities. However, the weighted proportion of cognitive impairment at the baseline was similar between respondents with stroke (18.03%; 95% CI, 13.95–22.12%) and those without stroke in the follow-up (17.74%; 95% CI, 16.25–19.24%).

**Table 2:** Weighted sample characteristics and comparisons stratified by stroke during the 8 years of follow-up^a^.

**Characteristic**	**Stroke**		
	**Yes (*N* = 416) (95% CI)**	**No (*N* = 5,828) (95% CI)**	***P^b^***
**Age groups, %, year**			0.0296
65–69	21.99 (16.59, 27.39)	30.05 (28.77, 31.32)	
70–74	28.95 (23.46, 34.45)	25.14 (24.08, 26.20)	
75–79	20.54 (16.19, 24.88)	19.19 (18.21, 20.16)	
80–84	13.82 (10.31, 17.33)	14.08 (13.32, 14.84)	
85–89	10.49 (7.63, 13.34)	8.01 (7.32, 8.69)	
≥90	4.21 (2.76, 5.66)	3.55 (3.14, 3.96)	
**Sex, %**			0.5658
Male	42.36 (36.92, 47.79)	44.02 (42.46, 45.58)	
Female	57.64 (52.21, 63.08)	55.98 (54.41, 57.54)	
**Race/ethnicity, %**			0.7609
White, non-hispanic	80.75 (75.73, 85.77)	82.16 (79.08, 85.24)	
Black, non-hispanic	9.23 (6.46, 11.99)	7.91 (6.11, 9.71)	
Hispanic	7.10 (3.33, 10.87)	6.66 (4.57, 8.74)	
Other	2.92 (0.83, 5.02)	3.28 (2.43, 4.12)	
**Educational level, %**			0.3638
No degree	21.60 (17.23, 25.97)	20.05 (17.90, 22.19)	
High school	34.29 (28.41, 40.16)	34.89 (32.61, 37.18)	
Some college	16.77 (12.00, 21.54)	13.83 (12.60, 15.06)	
College degree and more	27.34 (21.16, 33.52)	31.22 (28.31, 34.14)	
**Medicare-Medicaid enrollees, %**			0.0205
Yes	14.91 (10.55, 19.28)	10.67 (9.18, 12.17)	
No	85.09 (80.72, 89.45)	89.33 (87.83, 90.82)	
**Proxy respondent, %**			0.5470
Yes	3.76 (1.70, 5.82)	4.45 (3.79, 5.12)	
No	96.24 (94.18, 98.30)	95.55 (94.88, 96.21)	
**Depression, %**			0.0657
Yes	8.20 (5.21, 11.18)	5.74 (4.92, 6.56)	
No	91.80 (88.82, 94.79)	94.26 (93.44, 95.08)	
**Anxiety, %**			0.0724
Yes	8.52 (6.12, 10.93)	6.57 (5.70, 7.43)	
No	91.48 (89.07, 93.88)	93.43 (92.57, 94.30)	
**Smoking status, %**			0.4861
Never	49.68 (43.90, 55.46)	47.48 (45.73, 49.23)	
Former	43.73 (38.05, 49.41)	44.13 (42.38, 45.88)	
Current	6.60 (3.94, 9.25)	8.39 (7.38, 9.41)	
Cognitive impairment, %	18.03 (13.95, 22.12)	17.74 (16.25, 19.24)	0.8933
Comorbidities, mean	3.92 (3.76, 4.07)	3.73 (3.68, 3.78)	0.0213
Body mass index, mean	33.69 (32.69,34.69)	32.75 (32.48,33.02)	0.0726

a *Excluding participants with stroke at the baseline (N = 808)*.

b *P-values are calculated by the Rao-Scott Chis-square test for categorical variables and the unpaired t-tests for continuous variables*.

**Figure 3:**
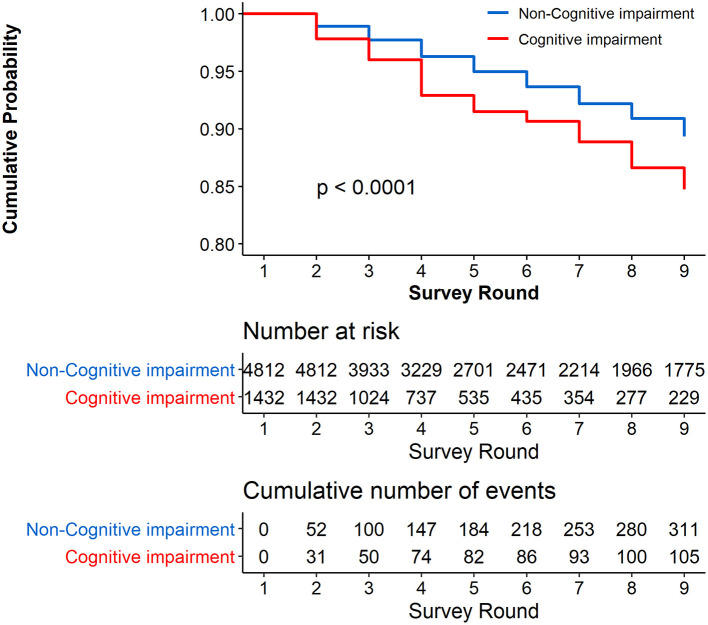
Cumulative Probability of not reporting stroke during the 8 years of follow-up. Respondents with stroke at the baseline were excluded (*N* = 808).

Figure 3 presents the Kaplan–Meier curves for cumulative probability of not reporting a stroke symptom in the follow-up among the respondents with and without cognitive impairment at the baseline. The cumulative proportion for participants with cognitive impairment at the baseline who did not report a stroke symptom was 84.73% (95% CI, 81.50–87.96%), and for those without cognitive impairment at the baseline who did not report a stroke symptom, the cumulative proportion was 89.33% (95% CI, 88.15–90.51%). The results show that the cumulative proportion of not reporting a stroke symptom was significantly higher in participants without cognitive impairment than in those with cognitive impairment at the baseline (*P* < 0.0001). Supplementary Table 3 shows that the unadjusted HR of cognitive impairment was 1.436 (95% CI, 1.088–1.896, *P* = 0.0111).

**Figure 4:**
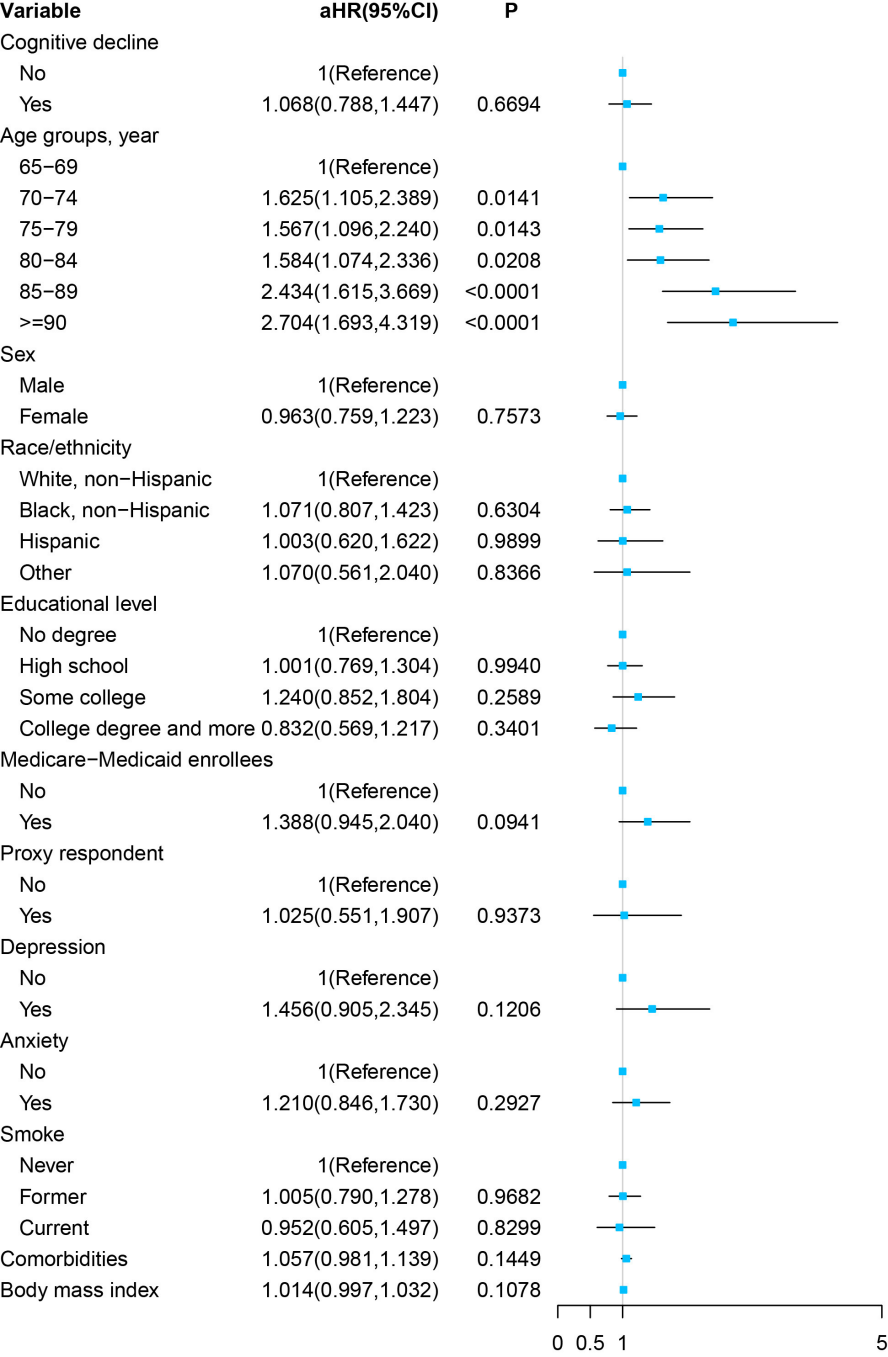
Forest plot of multivariable proportional hazards regression modeling the development of stroke over 8 years of follow-up.

As Figure 4 and Supplementary Table 4 show, in the multivariable Cox proportional hazards regression model, adjusted by the age, sex, race/ethnicity, educational level, Medicare–Medicaid eligibility, proxy respondent, depression, anxiety, smoking status, comorbidities, and BMI, cognitive impairment at the baseline was not significantly associated with a stroke symptom in the future (aHR, 1.068; 95% CI, 0.788–1.447, *P* = 0.6694).

Furthermore, in the sensitivity analyses, after excluding the data from proxy respondents, we reran the previous analysis. The results were similar to our main findings and are shown in Supplementary Tables 5, 6 and Supplementary Figures 1, 2.

## Discussion

Based on the representative sample of community-dwelling elders 65 years old from the NHATS, our study found a significant association between baseline stroke and subsequent cognitive impairment incidence; and the reverse relation was not true after adjusting for potential confounders, although the crude HR was significant. To our best knowledge, this is the first time to evaluate the longitudinal bidirectional association between stroke and cognitive impairment in the United States.

A number of studies have demonstrated that history of stroke could increase the risk of cognitive impairment in both the United States (9–12) and other countries outside (13–18) consistently. However, the past studies might be limited in representative of older adults in the United States with small sample size and outdated data. In comparison, our study provided the latest evidence on the association between baseline stroke and future cognitive impairment among old adults in the United States. In 2014, a study composed of four large USA cohort studies [the Cardiovascular Health Study (CHS); the Framingham Heart Study (FHS); the Health and Retirement Study (HRS); and the Sacramento Area Latino Study on Aging (SALSA)] including up to 20,229 participants until 2010 identified prevalent stroke as an independent indicator of cognitive impairment with pooled HR of 1.86 (12). In a meta-analysis conducted in 2018, the pooled HR of past stroke with future cognitive impairment was 1.69 (95% CI, 1.49–1.92) (29). The effect value observed in the current study was not strong enough with HR of 1.241 (95% CI, 1.011–1.522), and the discrepancy might be ascribed to different demographic profiles especially the relatively old age composition of population. Study finding again emphasized that prevalent stroke could elevate risk of cognitive impairment in adults 65 and older in the United States. More attention should be paid to prevent the incidence of cognitive impairment with the goal to reduce the heavy burden of disability and hospitalization from cognitive impairment among older adults in the United States (30, 31). Based on the findings, it is necessary to screen and take early intervention for cognitive impairment in the stroke population and transform modifiable risk factors (e.g., smoking, physical inactivity, and hypertension) (32) for stroke prevention in the general population.

The mechanisms of history of stroke with higher risk of developing cognitive impairment remain unclear. However, some studies provided underlying insights. Post-stroke cognitive impairment tends to occur after extensive cerebral infarction including large infarcts and multiple infarcts and disruption of brain regions along the frontal subcortical circuits with cognitive function (33). Chronic cerebral pathological changes named white matter changes (WMCs) and brain atrophy observed in neuroimaging studies were frequently related to stroke injury and increased the risk of different levels of cognitive impairment (34). Besides, stroke promotes the accumulation of amyloid plaques, thereby triggering a neurodegenerative process and subsequent conversion to cognitive impairment (35). Moreover, new data from human and animal studies have linked chronic cerebral inflammation caused by weakening clearance of myelin debris and a prolonged innate and adaptive immune response with cognitive impairment (36).

By contrast, evidence on the association between baseline cognitive impairment with subsequent stroke risk is sparse, and the results are discordant (19–23). Only a few studies were conducted in the United States. One study published in 1996 found that severe cognitive impairment preceded the incidence of stroke with a relative risk of 2.2 (95% CI, 1.2–3.8) in population aged 65 years and older (19), while another study consisting 48–67 years' population did not find any significant association between cognitive test results and ischemic stroke incidence (21). This is the first time that the association between diagnosis of cognitive impairment and future stroke risk in a nationally representative sample was examined, and we found that cognitive impairment was related to increased risk of stroke with crude HR of 1.436 (95% CI, 1.088–1.896) in univariate analysis. However, the association was diminished and no longer significant after adjustments for potential confounders, and age was the only significant predictor. This might be partially explained by fact that the effect of cognitive impairment on stroke was mainly reflected on the age, as the incidence of cognitive impairment and stroke increases with age in common (7, 8). Additionally, Alzheimer's disease (AD) is the most common subtype of dementia (37), and AD has been demonstrated as a risk factor of hemorrhagic stroke rather than ischemic stroke in previous studies (38–40). However, our study could not analyze the risk of developing stroke in cognitive impairment patients in specific subtypes due to the lack of relevant data. Along this line, the association between baseline cognitive impairment and stroke risk should be verified in future representative samples of old adults in the United States according to subtypes of cognitive impairment and stroke.

This study has some strengths. First, we used a nationally representative study of adults aged 65+ to evaluate the bidirectional association between stroke and cognitive impairment, which provided the theory reference for the prevention of stroke and cognitive impairment among old adults in the United States. Second, our study had high response rates of >80%, so the selection bias was alleviated. Third, long follow-up time with nine rounds of data ensured the sufficient incident cases and enough statistical power. Fourth, stroke and cognitive impairment were assessed by the same protocol each round with good internal measurement consistency.

Our study also has some limitation. First, evaluation of stroke and cognitive impairment was based on self-report rather than clinical diagnosis, which brought recall bias and misclassification. Second, not all possible confounders were unadjusted due to limited variables collected. Third, the bidirectional association between stroke and cognitive impairment could not be assessed according to specific subtypes, which should be discussed further in the future. Fourth, it is impossible to determine causality from current data, and relevant mechanisms should be further explored.

## Conclusion

In conclusion, this study examined the longitudinal bidirectional association between self-reported stroke and cognitive impairment in a nationally representative sample of older US adults and found that baseline stroke was independently associated with higher risk of developing cognitive impairment, while the inverse association was insignificant. The findings from this study may contribute to design and implementation of prevention and early interventions to reduce the burden of stroke and cognitive impairment in a rapidly aging population in the United States.

## Data Availability Statement

The original contributions presented in the study are included in the article/Supplementary Material, further inquiries can be directed to the corresponding author/s.

## Author Contributions

XW collected the data and drafted the manuscript. LF helped conceive the study and drafted the manuscript. SK, YH, and KZ contributed to language control and revised the manuscript. SY conceived the study, coordinated the study tasks, and helped draft the manuscript. All authors contributed to improvement of the manuscript and approved the submitted version.

## Conflict of Interest

KZ was employed by the company Biostatistician at Causality Clinical Data Technology Co., Ltd. The remaining authors declare that the research was conducted in the absence of any commercial or financial relationships that could be construed as a potential conflict of interest.
