# Corrigendum: Longitudinal Associations of Stroke With Cognitive Impairment Among Older Adults in the United States: A Population-Based Study

**DOI:** 10.3389/fpubh.2021.760605

**Published:** 2021-09-09

**Authors:** Xia Wu, Li Fan, Songqing Ke, Yangting He, Ke Zhang, Shijun Yang

**Affiliations:** ^1^Department of Otorhinolaryngology, Union Hospital, Tongji Medical College, Huazhong University of Science and Technology, Wuhan, China; ^2^Department of Orthopaedics, Union Hospital, Tongji Medical College, Huazhong University of Science and Technology, Wuhan, China; ^3^Ministry of Education Key Laboratory of Environment and Health, Department of Epidemiology and Biostatistics, School of Public Health, Tongji Medical College, Huazhong University of Science and Technology, Wuhan, China; ^4^Biostatistician at Causality Clinical Data Technology Co., Ltd, Wuhan, China; ^5^Department of Cardiology, Union Hospital, Tongji Medical College, Huazhong University of Science and Technology, Wuhan, China

**Keywords:** stroke, cognitive decline, older, longitudinal analysis, United States

In the published article, there was an error in affiliations 1, 2, 5. Instead of “Tongji Medical College, Union Hospital, Huazhong University of Science and Technology, Wuhan, China,”, it should be “Union Hospital, Tongji Medical College, Huazhong University of Science and Technology, Wuhan, China”.

The authors apologize for this error and state that this does not change the scientific conclusions of the article in any way. The original article has been updated.

## Publisher's Note

All claims expressed in this article are solely those of the authors and do not necessarily represent those of their affiliated organizations, or those of the publisher, the editors and the reviewers. Any product that may be evaluated in this article, or claim that may be made by its manufacturer, is not guaranteed or endorsed by the publisher.

