# Supplementary material for: Longitudinal Associations of Stroke With Cognitive Impairment Among Older Adults in the United States: A Population-Based Study
Source: Front Public Health. 2021 May 19;9:637042. doi: 10.3389/fpubh.2021.637042 (PMC8170040; doi:10.3389/fpubh.2021.637042)
Supplement: Supplementary file 1 [file Table_1.docx]

**Supplementary Table 1.** Univariate Cox proportional hazards regression results modeling the development of cognitive impairment over 8 years of follow-up^a^.

| Variable | HR^b^ (95%CI) | P |
| --- | --- | --- |
| Stroke |  |  |
| No | 1 (Reference) |  |
| Yes | 1.746 (1.461,2.088) | <0.0001 |
| Age groups, year |  |  |
| 65-69 | 1 (Reference) |  |
| 70-74 | 1.852 (1.469,2.334) | <0.0001 |
| 75-79 | 2.558 (2.091,3.130) | <0.0001 |
| 80-84 | 3.617 (2.903,4.507) | <0.0001 |
| 85-89 | 5.271 (4.255,6.528) | <0.0001 |
| ≧90 | 6.620 (4.965,8.826) | <0.0001 |
| Sex |  |  |
| Male | 1 (Reference) |  |
| Female | 1.041 (0.922,1.177) | 0.5123 |
| Race/ethnicity |  |  |
| White, non-Hispanic | 1 (Reference) |  |
| Black, non-Hispanic | 1.708 (1.503,1.942) | <0.0001 |
| Hispanic | 2.085 (1.614,2.692) | <0.0001 |
| Other | 1.340 (0.947,1.896) | 0.0974 |
| Educational level |  |  |
| No degree | 1 (Reference) |  |
| High school | 0.563 (0.486,0.652) | <0.0001 |
| Some college | 0.428 (0.344,0.534) | <0.0001 |
| College degree and more | 0.350 (0.301,0.407) | <0.0001 |
| Medicare-Medicaid enrollees |  |  |
| No | 1 (Reference) |  |
| Yes | 2.020 (1.710,2.386) | <0.0001 |
| Proxy respondent |  |  |
| No | 1 (Reference) |  |
| Yes | 3.902 (2.894,5.261) | <0.0001 |
| Depression |  |  |
| No | 1 (Reference) |  |
| Yes | 1.961 (1.589,2.419) | <0.0001 |
| Anxiety |  |  |
| No | 1 (Reference) |  |
| Yes | 2.196 (1.823,2.644) | <0.0001 |
| Smoke |  |  |
| Never | 1 (Reference) |  |
| Former | 0.908 (0.803,1.026) | 0.1196 |
| Current | 0.988 (0.769,1.268) | 0.9928 |
| Comorbidities | 1.125 (1.084,1.167) | <0.0001 |
| Body mass index | 0.988 (0.980,0.996) | 0.0024 |

^a^, Participants with cognitive impairment at baseline were excluded (N=1762).

^b^, HR, hazards ratio.

**Supplementary Table 2.** Multivariable Cox proportional hazards regression results modeling the development of cognitive impairment over 8 years of follow-up^a^ (N=5290).

| Variable | aHR^b^ (95%CI) | *P* |
| --- | --- | --- |
| Stroke |  |  |
| No | 1 (Reference) |  |
| Yes | 1.241 (1.011,1.522) | 0.0387 |
| Age groups, year |  |  |
| 65-69 | 1 (Reference) |  |
| 70-74 | 1.756 (1.390,2.218) | <0.0001 |
| 75-79 | 2.512 (2.035,3.102) | <0.0001 |
| 80-84 | 3.523 (2.800,4.433) | <0.0001 |
| 85-89 | 5.118 (4.001,6.546) | <0.0001 |
| ≧90 | 6.306 (4.719,8.425) | <0.0001 |
| Sex |  |  |
| Male | 1 (Reference) |  |
| Female | 0.838 (0.739,0.950) | 0.0064 |
| Race/ethnicity |  |  |
| White, non-Hispanic | 1 (Reference) |  |
| Black, non-Hispanic | 1.633 (1.419,1.879) | <0.0001 |
| Hispanic | 1.709 (1.372,2.129) | <0.0001 |
| Other | 1.260 (0.861,1.845) | 0.2318 |
| Educational level |  |  |
| No degree | 1 (Reference) |  |
| High school | 0.714 (0.621,0.821) | <0.0001 |
| Some college | 0.605 (0.491,0.747) | <0.0001 |
| College degree and more | 0.525 (0.449,0.613) | <0.0001 |
| Medicare-Medicaid enrollees |  |  |
| No | 1 (Reference) |  |
| Yes | 1.224 (1.027,1.459) | 0.0244 |
| Proxy respondent |  |  |
| No | 1 (Reference) |  |
| Yes | 2.158 (1.479,3.148) | 0.0001 |
| Depression |  |  |
| No | 1 (Reference) |  |
| Yes | 1.368 (1.083,1.728) | 0.0091 |
| Anxiety |  |  |
| No | 1 (Reference) |  |
| Yes | 1.674 (1.341,2.091) | <0.0001 |
| Smoking status |  |  |
| Never | 1 (Reference) |  |
| Former | 0.965 (0.864,1.079) | 0.5297 |
| Current | 1.150 (0.878,1.506) | 0.3062 |
| Comorbidities | 1.046 (1.006,1.088) | 0.0250 |
| Body mass index | 0.996 (0.988,1.004) | 0.2988 |

^a^, Participants with cognitive impairment at baseline were excluded (N=1762).

^b^, aHR, adjusted hazard ratio.

**Supplementary Table 3.** Univariate Cox proportional hazards regression results modeling the development of stroke over 8 years of follow-up^a^.

| Variable | HR^b^ (95%CI) | *P* |
| --- | --- | --- |
| Cognitive impairment |  |  |
| No | 1 (Reference) |  |
| Yes | 1.436 (1.088,1.896) | 0.0111 |
| Age groups, year |  |  |
| 65-69 | 1 (Reference) |  |
| 70-74 | 1.656 (1.132,2.423) | 0.0099 |
| 75-79 | 1.589 (1.100,2.294) | 0.0141 |
| 80-84 | 1.584 (1.076,2.333) | 0.0203 |
| 85-89 | 2.403 (1.623,3.559) | <0.0001 |
| ≧90 | 2.663 (1.701,4.168) | <0.0001 |
| Sex |  |  |
| Male | 1 (Reference) |  |
| Female | 1.059 (0.852,1.316) | 0.6039 |
| Race/ethnicity |  |  |
| White, non-Hispanic | 1 (Reference) |  |
| Black, non-Hispanic | 1.269 (1.017,1.582) | 0.0348 |
| Hispanic | 1.265 (0.817,1.960) | 0.2884 |
| Other | 1.088 (0.586,2.021) | 0.7868 |
| Educational level |  |  |
| No degree | 1 (Reference) |  |
| High school | 0.826 (0.651,1.048) | 0.1141 |
| Some college | 0.960 (0.662,1.391) | 0.8280 |
| College degree and more | 0.611 (0.433,0.862) | 0.0054 |
| Medicare-Medicaid enrollees |  |  |
| No | 1 (Reference) |  |
| Yes | 1.696 (1.245,2.309) | 0.0010 |
| Proxy respondent |  |  |
| No | 1 (Reference) |  |
| Yes | 1.563 (0.895,2.731) | 0.1156 |
| Depression |  |  |
| No | 1 (Reference) |  |
| Yes | 1.810 (1.215,2.694) | 0.0039 |
| Anxiety |  |  |
| No | 1 (Reference) |  |
| Yes | 1.552 (1.135,2.122) | 0.0063 |
| Smoke |  |  |
| Never | 1 (Reference) |  |
| Former | 0.987 (0.779,1.252) | 0.9154 |
| Current | 0.890 (0.567,1.395) | 0.6073 |
| Comorbidities | 1.119 (1.036,1.210) | 0.0047 |
| Body mass index | 1.011 (0.995,1.028) | 0.1808 |

^a^, Participants with stroke at baseline were excluded (N=808).

^b^, HR, hazard ratio.

**Supplementary Table 4**. Multivariable Cox proportional hazards regression results modeling the development of stroke over 8 years as a function of cognitive impairment at the baseline^a^ (N=6244).

| Variable | aHR^b^ (95%CI) | *P* |
| --- | --- | --- |
| Cognitive impairment |  |  |
| No | 1 (Reference) |  |
| Yes | 1.068 (0.788,1.447) | 0.6694 |
| Age groups, year |  |  |
| 65-69 | 1 (Reference) |  |
| 70-74 | 1.625 (1.105,2.389) | 0.0141 |
| 75-79 | 1.567 (1.096,2.240) | 0.0143 |
| 80-84 | 1.584 (1.074,2.336) | 0.0208 |
| 85-89 | 2.434 (1.615,3.669) | <0.0001 |
| ≧90 | 2.704 (1.693,4.319) | <.00001 |
| Sex |  |  |
| Male | 1 (Reference) |  |
| Female | 0.963 (0.759,1.223) | 0.7573 |
| Race/ethnicity |  |  |
| White, non-Hispanic | 1 (Reference) |  |
| Black, non-Hispanic | 1.071 (0.807,1.423) | 0.6304 |
| Hispanic | 1.003 (0.620,1.622) | 0.9899 |
| Other | 1.070 (0.561,2.040) | 0.8366 |
| Educational level |  |  |
| No degree | 1 (Reference) |  |
| High school | 1.001 (0.769,1.304) | 0.9940 |
| Some college | 1.240 (0.852,1.804) | 0.2589 |
| College degree and more | 0.832 (0.569,1.217) | 0.3401 |
| Medicare-Medicaid enrollees |  |  |
| No | 1 (Reference) |  |
| Yes | 1.388 (0.945,2.040) | 0.0941 |
| Proxy respondent |  |  |
| No | 1 (Reference) |  |
| Yes | 1.025 (0.551,1.907) | 0.9373 |
| Depression |  |  |
| No | 1 (Reference) |  |
| Yes | 1.456 (0.905,2.345) | 0.1206 |
| Anxiety |  |  |
| No | 1 (Reference) |  |
| Yes | 1.210 (0.846,1.730) | 0.2927 |
| Smoke |  |  |
| Never | 1 (Reference) |  |
| Former | 1.005 (0.790,1.278) | 0.9682 |
| Current | 0.952 (0.605,1.497) | 0.8299 |
| Comorbidities | 1.057 (0.981,1.139) | 0.1449 |
| Body mass index | 1.014 (0.997,1.032) | 0.1078 |

^a^, Participants with stroke at baseline were excluded (N=808).

^b^, aHR, adjusted hazard ratio.

**Supplementary Table 5.** Multivariable Cox proportional hazards regression results modeling the development of cognitive impairment over 8 years as a function of stroke at the baseline^a^ (N=5204).

| Variable | aHR^b^(95%CI) | *P* |
| --- | --- | --- |
| Stroke |  |  |
| No | 1 (Reference) |  |
| Yes | 1.261 (1.032,1.540) | 0.0236 |
| Age groups, year |  |  |
| 65-69 | 1 (Reference) |  |
| 70-74 | 1.745 (1.384,2.199) | <0.0001 |
| 75-79 | 2.511 (2.031,3.104) | <0.0001 |
| 80-84 | 3.537 (2.799,4.469) | <0.0001 |
| 85-89 | 5.208 (4.096,6.622) | <0.0001 |
| ≧90 | 6.742 (5.002,9.089) | <0.0001 |
| Sex |  |  |
| Male | 1 (Reference) |  |
| Female | 0.847 (0.746,0.963) | 0.0117 |
| Race/ethnicity |  |  |
| White, non-Hispanic | 1 (Reference) |  |
| Black, non-Hispanic | 1.638 (1.420,1.890) | <0.0001 |
| Hispanic | 1.756 (1.415,2.180) | <0.0001 |
| Other | 1.298 (0.850,1.980) | 0.2246 |
| Educational level |  |  |
| No degree | 1 (Reference) |  |
| High school | 0.712 (0.619,0.819) | <0.0001 |
| Some college | 0.609 (0.493,0.753) | <0.0001 |
| College degree and more | 0.526 (0.448,0.616) | <0.0001 |
| Medicare-Medicaid enrollees |  |  |
| No | 1 (Reference) |  |
| Yes | 1.234 (1.028,1.482) | 0.0243 |
| Depression |  |  |
| No | 1 (Reference) |  |
| Yes | 1.416 (1.116,1.797) | 0.0045 |
| Anxiety |  |  |
| No | 1 (Reference) |  |
| Yes | 1.682 (1.346,2.101) | <0.0001 |
| Smoke |  |  |
| Never | 1 (Reference) |  |
| Former | 0.971 (0.868,1.086) | 0.6057 |
| Current | 1.148 (0.870,1.514) | 0.3253 |
| Comorbidities | 1.043 (1.002,1.087) | 0.0410 |
| Body mass index | 0.995 (0.987,1.003) | 0.2367 |

^a^, Participants with cognitive impairment at baseline were excluded. And we exclude the data from proxy respondents (N=86).

^b^, aHR, adjusted hazard ratio.


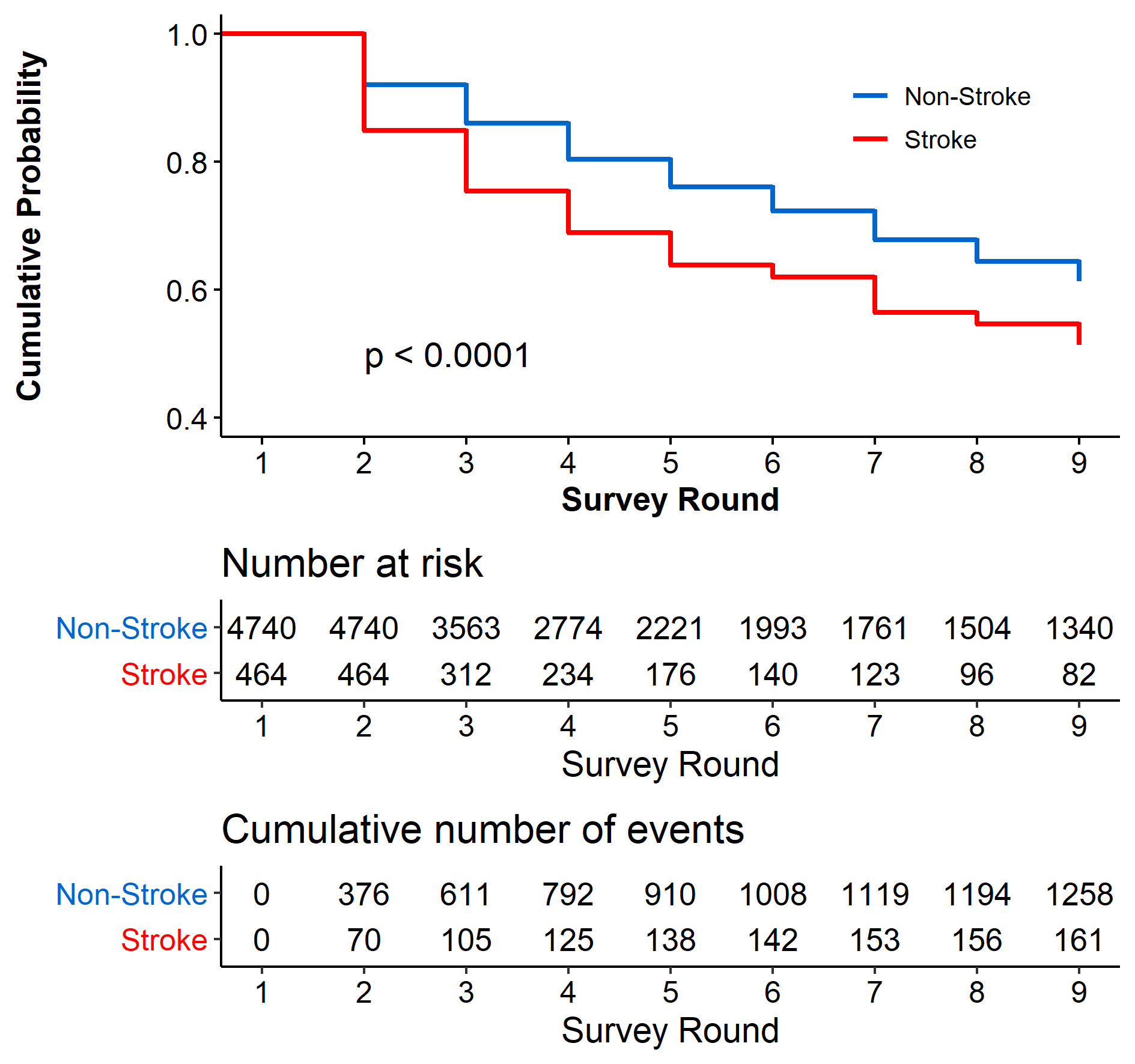


**Supplementary Figure 1.** Cumulative Probability of not reporting cognitive impairment during the 8 years of follow-up.

Respondents with cognitive impairment at the baseline were excluded. The data from proxy respondents was also excluded (N=86).

**Supplementary Table 6.** Multivariable Cox proportional hazards regression results modeling the development of stroke over 8 years as a function of cognitive impairment at the baseline^a^ (N=5833).

| Variable | aHR^b^(95%CI) | *P* |
| --- | --- | --- |
| Cognitive impairment |  |  |
| No | 1 (Reference) |  |
| Yes | 1.041 (0.761,1.425) | 0.7980 |
| Age groups, year |  |  |
| 65-69 | 1 (Reference) |  |
| 70-74 | 1.647 (1.117,2.429) | 0.0123 |
| 75-79 | 1.556 (1.082,2.238) | 0.0176 |
| 80-84 | 1.562 (1.057,2.309) | 0.0257 |
| 85-89 | 2.351 (1.539,3.592) | 0.0001 |
| ≧90 | 2.847 (1.764,4.596) | <0.0001 |
| Sex |  |  |
| Male | 1 (Reference) |  |
| Female | 0.982 (0.763,1.263) | 0.8852 |
| Race/ethnicity |  |  |
| White, non-Hispanic | 1 (Reference) |  |
| Black, non-Hispanic | 1.043 (0.778,1.399) | 0.7742 |
| Hispanic | 0.981 (0.589,1.634) | 0.9406 |
| Other | 1.255 (0.656,2.402) | 0.4897 |
| Educational level |  |  |
| No degree | 1 (Reference) |  |
| High school | 1.003 (0.763,1.32) | 0.9822 |
| Some college | 1.244 (0.860,1.801) | 0.2435 |
| College degree and more | 0.799 (0.542,1.178) | 0.2543 |
| Medicare-Medicaid enrollees |  |  |
| No | 1 (Reference) |  |
| Yes | 1.483 (1.007,2.184) | 0.0461 |
| Depression |  |  |
| No | 1 (Reference) |  |
| Yes | 1.439 (0.88,2.354) | 0.1451 |
| Anxiety |  |  |
| No | 1 (Reference) |  |
| Yes | 1.323 (0.938,1.867) | 0.1098 |
| Smoke |  |  |
| Never | 1 (Reference) |  |
| Former | 1.020 (0.796,1.306) | 0.8765 |
| Current | 0.902 (0.566,1.435) | 0.6596 |
| Comorbidities | 1.049 (0.971,1.133) | 0.2269 |
| Body mass index | 1.014 (0.997,1.032) | 0.1071 |

^a^, Participants with stroke at baseline were excluded. And we exclude the data from proxy respondents (N=361).

^b^, aHR, adjusted hazard ratio.


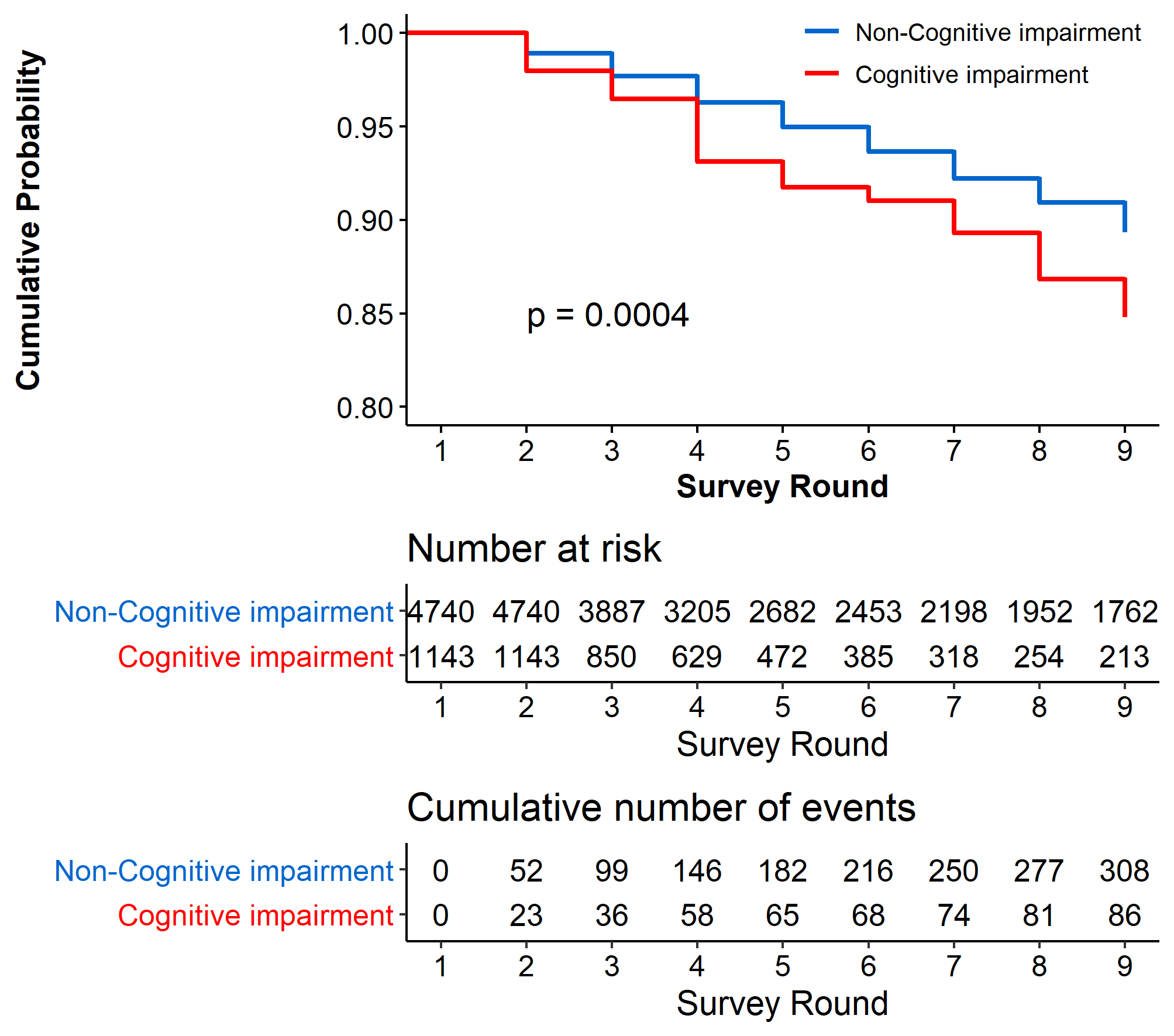


**Supplementary Figure 2.** Cumulative Probability of not reporting stroke during the 8 years of follow-up.

Respondents with stroke at the baseline were excluded. The data from proxy respondents was also excluded (N=361).
